# Cell-Free DNA as a Prognostic Biomarker for Monitoring Muscle-Invasive Bladder Cancer

**DOI:** 10.3390/ijms231911732

**Published:** 2022-10-03

**Authors:** Raquel Carrasco, Mercedes Ingelmo-Torres, Ascensión Gómez, Ramón Trullas, Fiorella L. Roldán, Tarek Ajami, Davinia Moreno, Leonardo Rodríguez-Carunchio, Antonio Alcaraz, Laura Izquierdo, Lourdes Mengual

**Affiliations:** 1 Laboratori i Servei d’Urologia, Hospital Clinic de Barcelona, 08036 Barcelona, Spain; 2 Genètica i Tumors Urològics, Institut d’Investigacions Biomèdiques August Pi i Sunyer (IDIBAPS), 08036 Barcelona, Spain; 3 Departament de Biomedicina, Facultat de Medicina i Ciències de la Salut, Universitat de Barcelona (UB), 08036 Barcelona, Spain; 4 Unitat de Neurobiologia, Institut d’Investigacions Biomèdiques de Barcelona (IIBB/CSIC/IDIBAPS), 08036 Barcelona, Spain; 5 Servei d’Anatomia Patològica, Hospital Clínic de Barcelona, 08036 Barcelona, Spain; 6 Facultat de Medicina, Universitat de Vic—Universitat Central de Catalunya (UVic-UCC), 08500 Vic, Spain; 7 Departament de Cirurgia i Especialitats Medicoquirúrgiques, Facultat de Medicina i Ciències de la Salut, Universitat de Barcelona (UB), 08036 Barcelona, Spain

**Keywords:** bladder cancer, cell-free DNA, circulating tumor DNA, droplet digital PCR, prognosis

## Abstract

Cell-free DNA (cfDNA) has recently emerged as a real-time biomarker for diagnosis, monitoring and prediction of therapy response in tumoral disease. Here, we evaluated cfDNA as a prognostic biomarker for monitoring muscle-invasive bladder cancer (MIBC) patients at different follow-up time points. Blood samples from 37 MIBC patients who underwent radical cystectomy (RC) were collected at cystectomy and 1, 4, 12 and 24 months later. Plasma cfDNA amount and fragmentation patterns were determined. Four mutations were analyzed in cfDNA to detect circulating tumor DNA (ctDNA) during patient follow-up. During a median follow-up of 36 months, 46% of patients progressed; median time to progression was 10 months. cfDNA levels and ctDNA status four months after RC were identified as independent prognostic biomarkers of tumor progression (HR 5.290; *p* = 0.033) and cancer-specific survival (HR 4.199; *p* = 0.038), respectively. Furthermore, ctDNA clearance four months after RC was significantly associated with patients’ clinical outcomes. In conclusion, cfDNA levels and ctDNA status four months after RC have prognostic implications in MIBC patients. In addition, cfDNA monitoring is useful to predict patient outcomes after RC. cfDNA analysis in the clinical setting could greatly improve MIBC patient management.

## 1. Introduction

Approximately 25% of bladder cancer (BC) patients are diagnosed with muscle-invasive bladder cancer (MIBC). Furthermore, about 40–50% of bladder cancer initially diagnosed as high-grade non-muscle invasive tumors will progress to MIBC during follow-up [1,2]. Radical cystectomy (RC) is the gold standard treatment for localized MIBC [1], although this treatment is also recommended for recurrent high-risk non-muscle-invasive bladder cancer (NMIBC) patients and NMIBC refractory, relapsing or unresponsive to intravesical bacillus Calmette–Guérin (BCG) treatment [1,3]. Neoadjuvant cisplatin-based chemotherapy is used in selected patients due to its benefits in survival, while other patients are candidates for adjuvant therapies to reduce tumor progression probabilities [1]. Unfortunately, despite all these treatments, around 50% of MIBC patients will develop local or distant metastasis within two years of RC [1,4].

The follow-up of BC patients after RC is currently performed using imaging techniques [1]; nonetheless, lesion size and imaging background could hinder the detection of tumor dissemination [5]. Consequently, an effective and non-invasive tool to provide early detection of tumor progression and treatment response would be very useful for monitoring BC after RC.

Cell-free DNA (cfDNA) has recently emerged as a real-time biomarker for diagnosis, monitoring and the prediction of therapy response in tumoral disease [6,7]. Primary and metastatic tissue samples are usually difficult to obtain, whereas cfDNA can be obtained in a minimally invasive way. cfDNA has a short half-life in blood, and several cfDNA samples can be obtained at different follow-up time points [8], making it optimal for real-time monitoring of cancer patients. High cfDNA levels [9,10,11,12] and short cfDNA fragments (between 100 and 150 bp deriving from the apoptosis of tumor cells [13]) in the bloodstream [14,15] have been proposed as biomarkers of poor prognosis in different solid tumors, including urologic tumors [12,16], but not in bladder cancer.

Circulating tumor DNA (ctDNA) corresponds to a low proportion of cfDNA that could be released from either primary tumor, metastasis or apoptotic circulating tumor cells [17]. ctDNA reproduces the somatic genome of tumor tissue; an 80% mutation concordance between ctDNA and matched tumor tissue has been described [18]. In bladder cancer, the presence of ctDNA in plasma has already been correlated with poor outcomes [19,20,21,22,23].

In this work, we aimed to ascertain whether cfDNA levels and fragmentation patterns as well as ctDNA status could be useful biomarkers to monitor MIBC patients after RC. Specifically, we evaluated cfDNA levels and fragmentation patterns as well as ctDNA status in MIBC patients after surgery at different follow-up time points and correlated them with the patients’ clinicopathological characteristics and clinical outcomes.

## 2. Results

### 2.1. Clinicopathological Features of the Cohort

The demographic and clinicopathological characteristics of patients enrolled in the study are shown in Table 1.

During a median follow-up of 36 months, 17 (46%) patients progressed (2 with LN+); median time to progression was 10 months (range 1–42 months). In all, 5 of the 17 patients who progressed received neoadjuvant or adjuvant chemotherapy; the remaining progressive patients were not fit for chemotherapy or did not consent to it. Overall, 82.4% (14/17) of progressive patients received treatment at progression. During follow-up, 17 (46%) patients died, 14 (82.4%) due to BC. Of these 14 patients, 9 (64%) had pT3 and pT4 tumors at BC diagnosis and 7% (1/14) had LN+. The median time of cancer-specific survival (CSS) was 16 months (range: 4–35 months). Three non-progressive patients developed another primary tumor during follow-up. These tumors were sarcoma, lung and ovarian cancers. As 17 (46%) patients died within 2 years of follow-up, we were unable to perform any statistical analysis including cfDNA samples collected 24 months after RC.

### 2.2. Mutation Analysis from Tissue and Plasma at Cystectomy

In tissue samples, *ATM*, *TP53* and *TERT* were the most frequently mutated genes in our cohort. Notably, the c.-124C>T hotspot mutation in the *TERT* promoter was found in 69% (25/36) of cases. *TERT* c.-146C>T, *ATM* c.1236-2A>T and *TP53* c.853G>A mutations were found in 16%, 42% and 13% of tissue samples, respectively. Interestingly, at least one of these four mutations was detected in 94% (34/36) of tissue samples [24].

**Figure 1:**
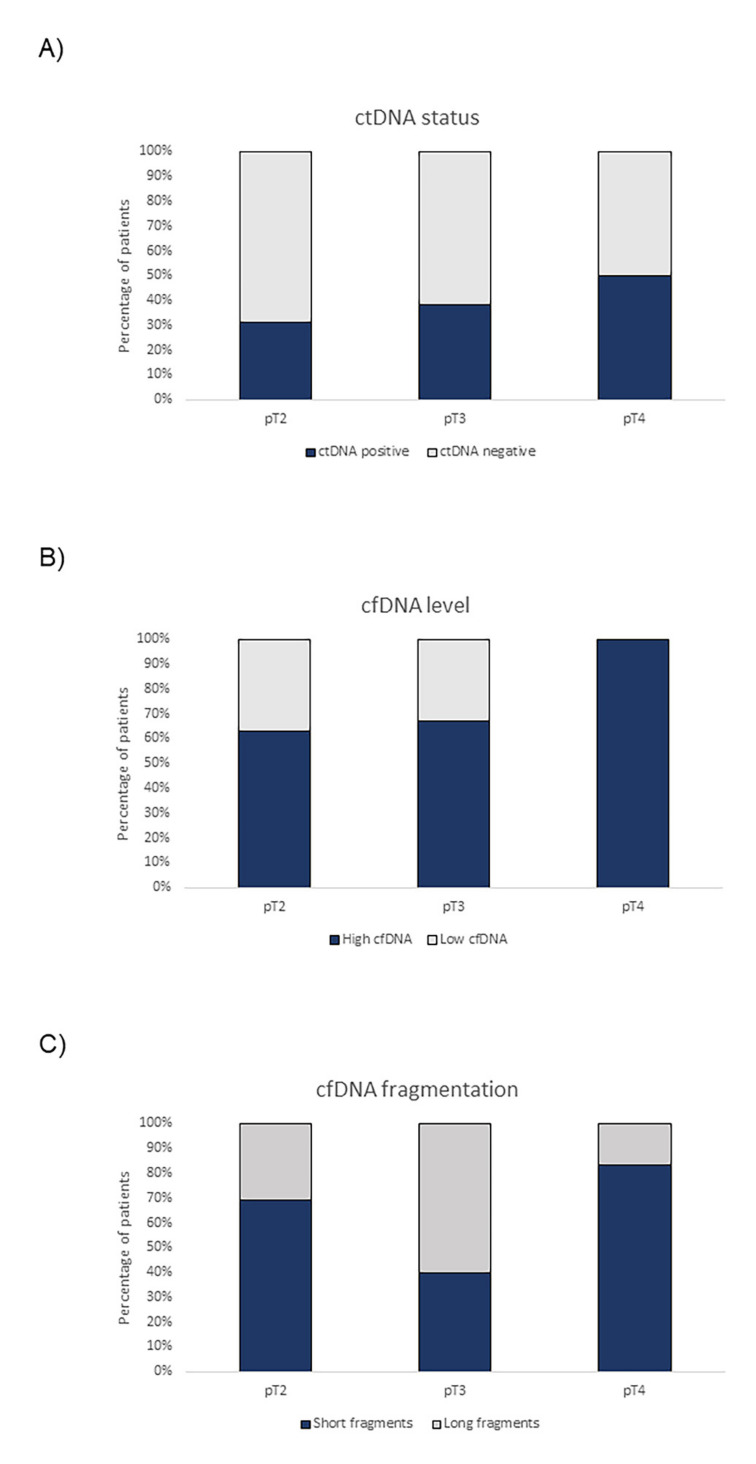
(**A**) ctDNA status, (**B**) cfDNA levels and (**C**) cfDNA fragmentation patterns at baseline according to the pathological state of MIBC patients.

When analyzing these four mutations in ctDNA at baseline, we found a 65% mutation concordance between ctDNA and matched tumor tissue. On the other hand, at least one of the four mutations was found in 66% (23/35) of cfDNA samples at cystectomy; *TERT* c.-124C>T, *TERT* c.-146C>T, *ATM* c.1236-2A>T and *TP53* c.853G>A mutations were found in 64% (16/25), 40% (2/5), 38% (6/16) and 100% (6/6) of ctDNA samples analyzed at baseline, respectively. At this time, ctDNA status and cfDNA levels were associated with the pathological stage of MIBC patients; the higher pT stage tended to present more ctDNA positive samples and higher cfDNA levels (Figure 1A,B). Contrarily, cfDNA fragmentation did not show any clear association with the pathological stage (Figure 1C).

### 2.3. ctDNA Status and cfDNA Levels and Fragmentation Patterns during Follow-Up

The mean mutant allele fraction (MAF) for each mutation, mean cfDNA levels and mean cfDNA fragmentation patterns during follow-up of MIBC patients were determined (Figure S1). Mean MAF of *TERT* c.-124C>T and *TP53* c.853G>A changed significantly during follow-up (*p* = 0.03 and *p* = 0.011, respectively). Similarly, mean cfDNA levels significantly changed during follow-up (*p* = 0.018), presenting the highest levels four months after RC, while no significant changes were found in mean cfDNA fragmentation.

**Figure 2:**
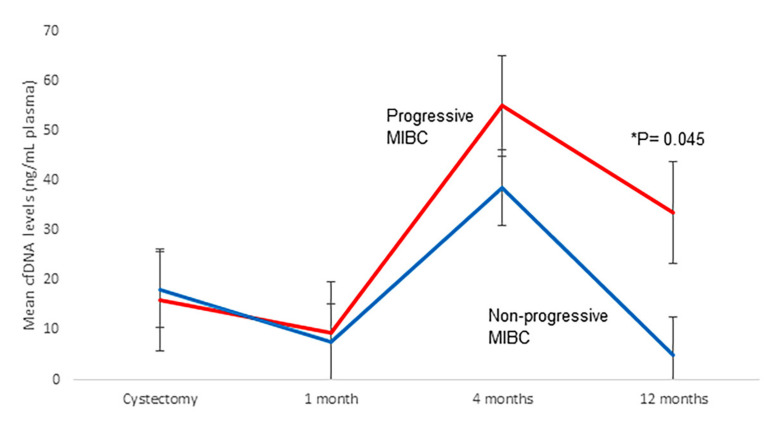
Analysis of cfDNA levels in MIBC patients according to tumor progression.

When dividing patients according to progression and death status, we found that the mean MAF of *TERT* c.-124C>T was significantly higher in progressive than in non-progressive MIBC patients and in those patients who died due to BC 4 and 12 months after RC (Figure S2). Furthermore, mean cfDNA levels 12 months after RC were significantly higher in progressive than in non-progressive MIBC patients (*p* = 0.045; Figure 2). No significant differences were found in the mean cfDNA fragment size of MIBC patients (Table S1) or mean MAF for the remaining mutations (Table S2) according to tumor progression and death occurrence.

### 2.4. Prognostic Value of ctDNA and cfDNA

**Figure 3:**
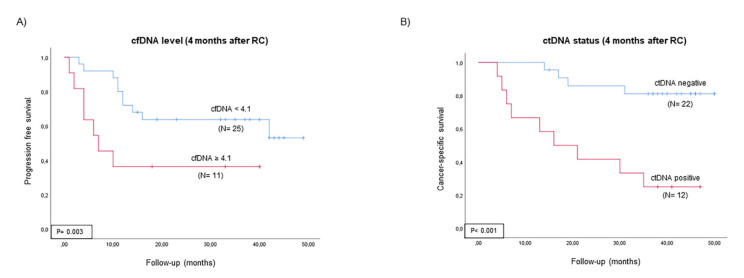
Prognostic value of ctDNA status and cfDNA level. Kaplan–Meier survival analysis shows probability of (**A**) tumor progression in MIBC patients stratified by cfDNA level and (**B**) CSS in MIBC stratified by ctDNA status at four months.

ctDNA status and cfDNA level as well as clinicopathological variables during patient follow-up for each patient are summarized in Figure S3. ctDNA status, cfDNA level and fragmentation pattern at each follow-up time point and clinicopathological variables were evaluated through Cox regression analysis. Multivariate Cox regression analysis identified cfDNA level four months after RC as an independent prognostic biomarker for tumor progression (HR 5.290; *p* = 0.033) and positive ctDNA status four months after RC as an independent biomarker for CSS (HR 4.199; *p* = 0.038). The median cfDNA level (4.1 ng/mL plasma) and ctDNA status (presence or absence) four months after RC were used as a cutoff point to classify patients into high- and low-risk groups for tumor progression and CSS. Figure 3 depicts the Kaplan–Meier curves generated using the selected cutoff points, showing that they could discriminate between two groups of MIBC patients with a significantly different probability of tumor progression and CSS after RC.

### 2.5. Detection of Tumor Progression According to Changes in ctDNA Status and cfDNA Level

Changes in ctDNA status and cfDNA levels to detect tumor progression compared to currently used radiological techniques were determined in each progressive patient.

**Figure 4:**
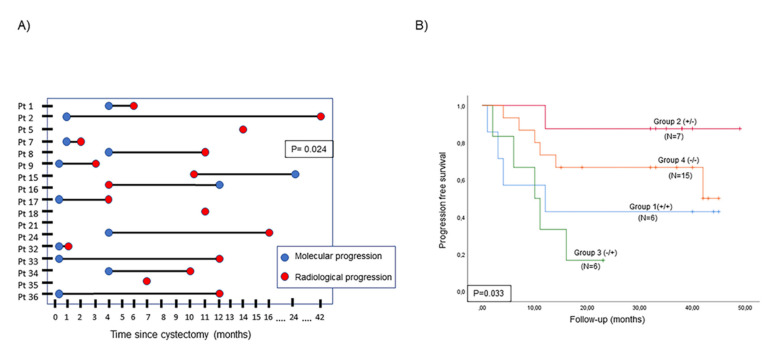
ctDNA status analysis. (**A**) Time of progression according to changes in ctDNA status (molecular progression) and imaging techniques (radiological progression); and (**B**) Kaplan–Meier curve for tumor progression in four different MIBC groups stratified by changes in ctDNA status between baseline and four months after RC (Group 1: patients with positive ctDNA status at both time points; Group 2: patients with positive ctDNA status at baseline and negative ctDNA status four months after RC; Group 3: patients with negative ctDNA status at baseline but positive ctDNA status after RC; Group 4: patients with negative ctDNA status at baseline and later). Abbreviations: Pt; patient.

Taking into account positive ctDNA status, the median anticipation period for molecular progression compared with radiological progression was 6 months (*p* = 0.024; Figure 4A).

On the other hand, high cfDNA levels were not able to predict tumor progression.

### 2.6. Prognostic Value of Changes in ctDNA Status and cfDNA Level

To determine the prognostic value of the changes in ctDNA status and cfDNA levels between cystectomy and four months later, patients were stratified into four groups: Group 1: patients with positive ctDNA status or high cfDNA levels at both time points; Group 2: patients with positive ctDNA status or high cfDNA levels at baseline and negative ctDNA status or low cfDNA levels four months after RC; Group 3: patients with negative ctDNA status or low cfDNA levels at baseline but positive ctDNA status or high cfDNA levels after RC; Group 4: patients with negative ctDNA status or low cfDNA levels at baseline and later. Changes in ctDNA status occurred between baseline and four months after RC indicated that Group 2 and Group 4 patients had a better prognosis (*p* = 0.033) and longer CSS (*p* = 0.002) than those in Group 1 and Group 3 (Figure 4B and Figure S4). Moreover, patients with positive ctDNA status only after RC (Group 3) tended to have a worse prognosis and shorter CSS than those with positive ctDNA status in all samples (Group 1) (Figure 4B and Figure S4). Regarding changes in cfDNA, no differences were found between the different groups (*p* > 0.05).

ctDNA clearance four months after RC was significantly associated with clinical outcomes (*p* = 0.033).

## 3. Discussion

In recent years, liquid biopsy has been studied in oncology as a tool for the real-time assessment and management of tumor evolution. It is less invasive, and samples are more easily obtainable throughout the disease’s course in comparison with tumor-tissue-based approaches [25]. cfDNA as well as the proportion of ctDNA present in the cfDNA have been considered useful biomarkers to predict the patient’s prognosis and treatment response in several solid tumors [9,10,26]. Of note, in lung, melanoma or gastroesophageal cancers, the presence of ctDNA in the blood is a well-established biomarker of response to targeted therapies in the clinical setting [26,27]. In BC, some studies have described the presence of ctDNA and its association with poor outcomes [19,20,21,22,23]; however, ctDNA is not currently being used in routine clinical practice in the follow-up of BC patients after RC. This is most likely due to the genetic heterogeneity of this tumor, presenting a challenge when it comes to defining a generic set of biomarkers to detect the presence of ctDNA in any single patient.

Here, in ctDNA samples, we analyzed four somatic mutations previously identified in 94% of patients from our cohort. We demonstrated a high mutation concordance between ctDNA and matched tumor tissue at baseline, as previously described [18]. We also found that ctDNA status at cystectomy directly correlated with a higher pathological stage in MIBC patients. In agreement with this result, Yang et al. [28] also reported that stages were closely associated with baseline ctDNA detection in lung cancer patients, finding that patients with positive ctDNA tended to have more advanced disease.

Interestingly, when analyzing the ctDNA monitoring of each mutation, we found that the mean MAF of *TERT* c.-124C>T 4 and 12 months after RC was significantly higher in progressive than in non-progressive patients and in those patients who died due to BC. Similarly, a high MAF of *TERT* c.-124C>T in urinary ctDNA was found to be associated with bladder tumor recurrence [29,30,31]. In addition, Pritchard et al. [22] detected *TERT* c.-124C>T in plasma and urine ctDNA of BC patients via ddPCR. Moreover, *TERT* c.-124C>T has also been found in other solid tumors, such as melanoma and hepatocellular carcinoma, and its presence in cfDNA has been associated with poor prognosis [32,33,34]. Overall, these findings suggest that the *TERT* c.-124C>T mutation could play a crucial role in the aggressiveness of tumors due to promoting alterations in telomerase activity and avoiding cell senescence [35]. Finally, we have demonstrated that patients with a positive ctDNA status four months after RC are at high risk of cancer-specific mortality, which further supports the prognostic utility of ctDNA analysis in the follow-up of MIBC patients undergoing RC. In inoperable lung cancer, Yang et al. [28] demonstrated that ctDNA positivity one month after therapy could estimate patient prognosis. Moreover, Tie et al. [36] found that colon cancer patients with detectable ctDNA after surgery had a high risk of recurrence.

In this work, we also determined plasma cfDNA levels and fragmentation patterns of MIBC patients who are candidates for RC. To the best of our knowledge, our study is the first to analyze plasma cfDNA in MIBC patients, correlating it with the patient’s outcome. Like ctDNA, cfDNA levels also increased during patient follow-up; the mean cfDNA level 12 months after RC was found to be significantly higher in progressive than in non-progressive patients. Furthermore, we have demonstrated that patients with high cfDNA levels four months after RC were at high risk of tumor progression. Toledano-Fonseca et al. [14] also demonstrated that in metastatic pancreatic cancer, high cfDNA levels were strongly associated with poor outcomes. By contrast, although cfDNA fragmentation size had been associated with tumor progression in several solid tumors [14,15], we did not find any correlation between cfDNA fragmentation and tumor progression or CSS in our series.

We also evaluated the anticipation period for detecting tumor progression of molecular techniques over conventional imaging techniques. In agreement with other studies [37,38], we demonstrated that ctDNA status could identify BC recurrence with a lower median lead time compared with imaging techniques. Moreover, our previous study [24] determined that circulating tumor cell (CTC) enumeration was able to detect early tumor progression in MIBC, further supporting the usefulness of liquid biopsy in monitoring MIBC patients after RC.

We assessed changes in ctDNA status at baseline and four months later. We showed that patients’ molecular response, understood as plasma ctDNA clearance four months after RC, was associated with better outcomes. Shohdy et al. [38] also demonstrated that ctDNA clearance after adjuvant therapy was associated with longer survival in advanced BC patients, and Magbanua et al. [39] found a similar result in breast cancer patients. Moreover, Yang et al. [28] found that lung cancer patients with negative ctDNA status had better outcomes than those with positive ctDNA status after therapy. By contrast, we found that the appearance of ctDNA in plasma four months after RC in those BC patients that were ctDNA-negative at baseline was associated with poor prognosis, emphasizing that the emergence of ctDNA after RC could be considered a sign of tumor aggressiveness. A similar result was obtained by Tie et al. [40], who found that ctDNA positivity after treatment in colon cancer patients was associated with poorer outcomes.

The strength of the present work lies in the fact that it is the first to combine four somatic mutations to analyze serial plasma ctDNA status from MIBC patients who are candidates for RC, as well as to determine serial plasma cfDNA levels and fragmentation patterns in the same cohort. Even though this is a first approach towards developing a generic set of biomarkers for the follow-up of MIBC patients after RC, this work demonstrated that it is possible to monitor MIBC patients using a set of gene mutations that are relatively common in BC. Further gene mutations must be identified to span the entire genetic heterogeneity of BC. On the other hand, the evaluation of cfDNA levels has several advantages over ctDNA detection: it can be analyzed in all patients independently of the genetic characteristics of their tumor, it is less labor-intensive and has a reduced cost. However, it should be taken into account that cfDNA levels can rise due to non-tumor-related causes, so cfDNA levels must be treated with caution. The detection of specific tumor mutations in ctDNA could complement this analysis. Lastly, the technology used for this analysis is easily transferable to a clinical setting since it is reasonably simple and usually available in diagnostic laboratories.

We must acknowledge some study limitations. First is the series size, although it should be considered that our series included a total of 165 samples analyzed. Second, a minor proportion of the patients (5%) did not harbor any of the four mutations identified; thus, we could not perform ctDNA analysis on these patients. Last, a final validation of our results in a larger and independent series is necessary to define the real role of cfDNA monitoring in MIBC patients after RC.

## 4. Materials and Methods

### 4.1. Patients and Samples

A total of 37 consecutive MIBC patients (median age (range) 71 years (51–85); 30 males, 7 females) who underwent RC and extended lymphadenectomy at our center between 2018 and 2019 were prospectively included. The exclusion criterion was the presence of another active neoplasm. Follow-up data were available for all patients. Tumor dissemination was controlled postoperatively via a computed tomography scan at three-month intervals for the first year, six-month intervals for the next two years and annually thereafter. Tumors were considered progressing when relapse or distant metastasis developed during follow-up. Progression and CSS times were measured from the date of RC to progression or BC-specific death or the final follow-up date in cases without progression.

One 10 mL EDTA tube of peripheral blood was collected before RC and at 1, 4, 12 and 24 months after surgery. Blood samples were stored at 4 °C until processed within the following 24 h.

All methods were carried out following relevant guidelines and regulations. All patients provided written informed consent (HCB/2013/8753) before being enrolled in the study, and the study was approved by the Clinical Research Ethics Committee of the Hospital Clinic of Barcelona (HCB/2018/0026).

### 4.2. Targeted Next-Generation Sequencing of Tissue Samples

A total of 161 of the most relevant cancer driver genes were previously analyzed by targeted next-generation sequencing (NGS) using the Ion Torrent Oncomine Comprehensive Assay v3 (Thermo Fisher Scientific, Waltham, MA, USA) [24] in BC tissue samples from 36 of the 37 patients included in this study. For one patient (Pt 10), a tissue sample was not available in our tumor biobank. Tissue samples were obtained from cystectomy specimens (N = 31), but in the case of pT0 in the cystectomy specimen, samples were obtained from transurethral resection of the bladder tumor (TURBT) presenting a muscle-invasive tumor (N = 5). In this previous study [24], the most frequent mutations found in tumor tissue samples from our cohort were *TERT* c.-124C>T (66%), *TERT* c.-146C>T (16%), *ATM* c.1236-2A>T (42%) and *TP53* c.853G>A (13%).

### 4.3. Blood Sample Procedures

Blood samples were centrifuged at 3500 rpm for 15 min at 4 °C to separate plasma, followed by plasma centrifugation at 16,000× *g* for 10 min at 4 °C to remove any remaining cells. Plasma samples were stored at −80 °C until cfDNA extraction.

cfDNA was extracted from 2 to 5 mL of plasma (depending on availability) using the QIAamp Circulating Nucleic Acid kit (Qiagen, Hilden, Germany), according to the manufacturer’s instructions. In order to determine the cfDNA level, cfDNA was quantified using the Quant-iT PicoGreen dsDNA kit (Thermo Fisher Scientific, Waltham, MA, USA), in accordance with the manufacturer’s instructions. To analyze the cfDNA fragment size, the High Sensitivity D1000 ScreenTape Assay was used in an Agilent 2200 TapeStation System (Agilent Technologies, Santa Clara, CA, USA), in accordance with the manufacturer’s instructions.

### 4.4. Droplet Digital PCR

Determination of the ctDNA status in each patient was performed by detecting the specific tumor mutations of each patient in their cfDNA samples via droplet digital PCR (ddPCR). A total of four somatic mutations previously identified in tumor tissue (TERT c.-124C>T, TERT c.-146C>T, ATM c.1236-2A>T and TP53 c.853G>A) were analyzed in a total of 167 plasma cfDNA samples from 37 BC patients at the time of RC and at four time points during the first two years of follow-up (1, 4, 12 and 24 months) to determine ctDNA status at each follow-up time point. ddPCR was performed using the QX200 Droplet Digital PCR system (Bio-Rad Laboratories, Hercules, CA, USA) following the manufacturer’s instructions.

No mutations were found in the tumor tissue of two patients, and therefore, they were excluded from this analysis; however, Pt 10, whose tumor tissue was not available, was included in this study. For each patient, only mutations identified in their tumor tissue were subsequently analyzed in their plasma cfDNA, except for Pt 10, in whom all four mutations were analyzed (Table S3).

Assays to detect and quantify the fractional abundance of point mutations and corresponding wild-type alleles were either custom-designed using Primer3 Input (version 4.1.0) or commercially obtained (Thermo Fisher Scientific) (Table S4). The optimal annealing temperature and limit of detection (LOD) were determined for each assay (Supplementary Methods). The MAF was calculated as the number of droplets positive with mutant amplicon, divided by total droplets positive with amplicon (wild-type and mutant). Further details of ddPCR experiments are described in Supplementary Methods. Data analysis was carried out using QuantaSoft Analysis Pro Software, version 1.0 (Bio-Rad Laboratories, Hercules, CA, USA).

### 4.5. Statistical Analysis

Differences in ctDNA status, cfDNA level and cfDNA fragmentation patterns at different follow-up time points were analyzed using the Mann–Whitney U-test for independent samples and the Friedman test for repeated measures. A ctDNA sample was considered positive (or detectable) if at least one of the four mutations had a MAF value higher than 30% of all MAF values. This 30% MAF value corresponded to 0.2 for *TERT* c.-124C>T, 0.14 for *TERT* c.-146C>T, 0.06 for *ATM* c.1236-2A>T and 0.35 for *TP53* c.853G>A. Using this definition, 33% (52/157) of all plasma samples were ctDNA positive. cfDNA level and fragment size were dichotomized using their median value. These median values (4.1 ng/mL plasma and 157 bp) were used as cutoffs to discriminate between high and low cfDNA and fragmentation levels. Using these cutoff values, 45% (75/165) of all plasma samples had high cfDNA values and 52% (85/165) low cfDNA fragmentation levels. Kaplan–Meier curves were generated and compared using log-rank tests. Cox regression analysis was performed on ctDNA status, cfDNA level and cfDNA fragmentation patterns at different follow-up time points and clinicopathological variables (pathological stage, lymph nodes status, age, chemotherapy) to examine their influence on tumor progression and CSS. Statistical significance was established at a *p*-value of 0.05. All analyses were carried out with the SPSS software package (IMB SPSS Statistics 25).

## 5. Conclusions

cfDNA levels and ctDNA status four months after RC are biomarkers of tumor progression and CSS, respectively, in MIBC patients who underwent RC. In addition, cfDNA monitoring is useful to predict patient outcomes after RC. Moreover, ctDNA status detected tumor progression earlier than imaging techniques. The implementation of cfDNA analysis in the clinical setting could have an impact on disease management since patients could benefit from early treatment.

## Figures and Tables

**Table 1:** Clinicopathological features of MIBC patients enrolled in the study.

	TOTAL MIBCN (% or Range)	Progressive MIBCN (% or Range)	Non-Progressive MIBC N (% or Range)
	**(N = 37)**	**(N = 17)**	**(N = 20)**
Gender			
Male	30 (81)	13 (76.4)	17 (85)
Female	7 (19)	4 (23.6)	3 (15)
Median Age (yr)	71 (51–85)	72 (51–85)	70 (59–83)
Pathological Stage			
<pT2	7 (19)	1 (5.9)	6 (30)
pT2	9 (24)	4 (23.6)	5 (25)
pT3	15 (41)	7 (41.2)	8 (40)
pT4	6 (16)	5 (29.4)	1 (5)
Lymph Nodes (LN)			
LN+	4 (11)	2 (11.8)	2 (10)
pT2	1 (2.7)	-	1 (5)
pT3	2 (5.4)	1 (5.9)	1 (5)
pT4	1 (2.7)	1 (5.9)	-
LN-	33 (89)	15 (88.2)	18 (90)
Neoadjuvant Chemotherapy	7 (19)	4 (23.6)	3 (15)
<pT2	2 (5.4)	1 (5.9)	1 (5)
pT2	-	-	-
pT3	3 (8.1)	1 (5.9)	2 (10)
pT4	2 (5.4)	2 (11.8)	-
Adjuvant Chemotherapy	6 (16)	1 (5.9)	5 (25)
<pT2	-	-	-
pT2	1 (2.7)	-	1 (5)
pT3	3 (8.1)	-	3 (15)
pT4	2 (5.4)	1 (5.9)	1 (5)

Abbreviations: MIBC; muscle-invasive bladder cancer, LN; lymph nodes.

## Data Availability

Data are available upon reasonable request.

## Supplementary Materials

The following supporting information can be downloaded at: https://www.mdpi.com/article/10.3390/ijms231911732/s1.
