# Supplementary material for: Cell-Free DNA as a Prognostic Biomarker for Monitoring Muscle-Invasive Bladder Cancer
Source: Int J Mol Sci. 2022 Oct 3;23(19):11732. doi: 10.3390/ijms231911732 (PMC9570339; doi:10.3390/ijms231911732)
Supplement: Supplementary file 1 [file ijms-23-11732-s001.zip › ijms-1920137-supplementary.pdf]

SUPPLEMENTARY METHODS

- <u>Determination of annealing temperature</u>

The CloneJET PCR Cloning kit (Thermo Fisher Scientific) was used for cloning each gene region containing the specific mutation as well as the wild-type allele, according to the manufacturer's protocol. Chemocompetent E. Coli DH5α derivates (New England Biolabs) were used to transform the cloning products following the High-Efficiency Transformation protocol (C2987H; New England Biolabs) with minor modifications. Briefly, 50 ng of the cloning product were mixed with Dh5α cells in a final volume of 70 µl and tubes were incubated for 20 min at 4ºC. Then, cells were heat shocked at 42ºC for 90 seconds and cooled on ice for 2 minutes. LB medium (350 µl) was added to the transformed bacteria and the mix was incubated for 2 hours at 37ºC with agitation. LB agar with 50 µg/µl of carbenicillin plates were used for seeding the transformed bacteria as the pJET1.2/blunt vector carried β-lactamase, an ampicillin resistance gene. The bacteria were incubated overnight at 37ºC. The isolated colonies grown on the carbenicillin plates were picked and inserted in a tube with 5 ml of LB 50 µg/µl carbenicillin medium, and then were incubated for 12 hours at 37ºC with agitation. QIAprep Spin Miniprep Kit (Qiagen) was used to isolate plasmids, according to the manufacturer's instructions.

- <u>Determination of limit of detection (LOD)</u>

An optimal annealing temperature was identified for each of the four genes. For each gene, 10,000 copies of the wild-type (wt) allele were mixed with 10,000 copies of the mutant allele, and droplet digital PCR (ddPCR) was performed with eight different annealing temperatures (range: 53ºC to 63ºC) to identify the

optimal temperature at which the wt and mutant droplets are separated enough. The optimal annealing temperature for each assay was: 60ºC for *TERT* c.-124C>T, 59ºC for *TERT* c.-146C>T, 56.5ºC for *ATM* and 57.5ºC for *TP53*. Then, we detected the LOD for each assay performing the ddPCR with 10,000 copies of wt allele and different copies of mutant allele as indicated in the following table:

Wild-types copies	Mutant copies
10,000	5,000
10,000	2,500
10,000	1,000
10,000	500
10,000	250
10,000	150
10,000	100
10,000	50
10,000	10
10,000	5
10,000	1
10,000	0

The LOD of the mutant allele was 0.08% for *TERT* c.-124C>T and *TERT* c.-146C>T, 0.05% for *ATM* c.1236-2A>T and 0.1% for *TP53* c.853G>A.

- <u>ddPCR</u>

The reactions for custom assays (*ATM* and *TERT* c.-124C>T) were performed with 11 µl SuperMix no dUTP (Bio-Rad), 0.5 µl forward + reverse primers (40

μM), 1.1 μl HEX probe (5 μM), 1.1 ul FAM probe (5 μM) and 8.3 μl cfDNA sample. The reactions for commercial assays (*TP53* and *TERT* c.-146C>T) were performed with 11 μl SuperMix no dUTP (Bio-Rad), 1.1 μl assay (20 μM), 1.6 μl H2O and 8.3 μl cfDNA sample. PCR was performed as follows; 95ºC for 10 min then 40 cycles (except for *TERT* c.-124C>T which was 50 cycles) of 94ºC for 30 seconds, the specific assay annealing temperature for 1 min, followed by 98ºC for 10 min, and a final hold at 4ºC. Each reaction was performed with a negative control (10,000 copies wt allele + 0 copy mutant allele) and positive control (10,000 copies wt allele + 10 copies mutant allele).

ctDNA was defined as detectable if the mutant allele fraction (MAF) was above the LOD of each mutant amplicon.

SUPPLEMENTARY TABLES

Table S1. Mean cfDNA fragment size (bp) of MIBC patients during follow-up according to tumor progression and death.

		Cystectomy (N= 37)	1 month (N= 37)	4 months (N= 38)	12 months (N= 34)
TUMOR PROGRESSION	Progressive	164.47	158.94	167.71	159.38
	Non-progressive	154.50	158.20	212.53	158.37
DEATH	Cancer-specific death	163.50	160.71	161.57	161.30
	Non-cancer-specific death	156.39	157.22	210.37	157.64

*There were no significant differences (p>0.05) between progressive and non-progressive patients as well as between cancer-specific death and non-cancer-specific death at each follow-up time point.

Table S2. Mean MAF of each gene between (A) progressive and non-progressive MIBC patients and (B) patients who died due to BC at the different follow-up time points.

A)

	**Cystectomy**		**1 month**		**4 months**		**12 months**	
	Progressive (N=17)	Non-Progressive (N= 20)	Progressive (N= 17)	Non-Progressive (N= 20)	Progressive (N= 17)	Non-Progressive (N= 19)	Progressive (N= 13)	Non-Progressive (N= 19)
TERT c.-124C>T	0.17* (N= 11)	0.19* (N= 13)	0.08* (N= 11)	0.07* (N= 13)	1.45 (N= 11)	0.05 (N= 13)	4.52 (N= 8)	0.07 (N= 13)
TERT c.-146C>T	0.01* (N= 2)	0.24* (N= 3)	0.01* (N= 2)	0.05* (N= 3)	0.01* (N= 2)	0.09* (N= 2)	0.05* (N= 2)	0.12* (N= 2)
ATM c.1236-2A>T	0.10* (N= 8)	0.13* (N= 8)	0.05* (N= 8)	0.01* (N= 8)	0.04* (N= 8)	0.01* (N= 7)	0.01* (N= 6)	0.04* (N= 7)
TP53 c.853G>A	0.90* (N= 2)	1.77* (N= 4)	0.33* (N= 2)	0.70* (N= 4)	1.86* (N= 2)	1.24* (N= 4)	1.50* (N= 2)	1.80* (N= 4)

*There were no significant differences (p>0.05) between progressive and non-progressive patients.

B)

	Cystectomy		1 month		4 months		12 months	
	Patients CSD (N= 14)	Patients non-CSD (N= 23)	Patients CSD (N= 14)	Patients non-CSD (N= 23)	Patients CSD (N= 14)	Patients non-CSD (N= 22)	Patients CSD (N= 10)	Patients non-CSD (N= 22)
TERT c.-124C>T	0.20* (N= 10)	0.18* (N= 14)	0.09* (N= 10)	0.08* (N= 14)	1.61 (N= 10)	0.05 (N= 14)	5.26 (N= 6)	0.07 (N= 14)
TERT c.-146C>T	0.03* (N= 1)	0.18* (N= 4)	0.02* (N= 1)	0.03* (N= 4)	0.02* (N= 1)	0.06* (N= 3)	0.05* (N= 1)	0.11* (N= 3)
ATM c.1236-2A>T	0.13* (N= 6)	0.10* (N= 10)	0.06* (N= 6)	0.01* (N= 10)	0.05* (N= 6)	0.01* (N= 9)	0.01* (N= 4)	0.04* (N= 9)
TP53 c.853G>A	0.90* (N= 2)	1.77* (N= 4)	0.33* (N= 2)	0.70* (N= 4)	1.86* (N= 2)	1.24* (N= 4)	1.50* (N= 2)	1.80* (N= 4)

Abbreviation: CSD; cancer-specific death

*There were no significant differences (p>0.05) between CSD and non-CSD at each follow-up time point.

Table S3. Plasma mutations analyzed in each patient.

	TERT c.-124C>T	*TERT* c.-146C>T	*ATM* c.1236-2A>T	*TP53* c.853G>A
Patient 1	X			
Patient 2	X			
Patient 3	X			
Patient 4	X			
Patient 5	X			
Patient 6	X			
Patient 7	X			
Patient 8			X	
Patient 9	X		X	
Patient 10	X	X	X	X
Patient 11	X			
Patient 12				X
Patient 13	X			
Patient 14		X	X	
Patient 15		X	X	
Patient 16			X	
Patient 17	X		X	
Patient 18		X		
Patient 19	X			
Patient 20		X	X	
Patient 21				
Patient 22	X			X
Patient 23			X	X
Patient 24	X			
Patient 25	X			
Patient 26				
Patient 27			X	
Patient 28	X			
Patient 29	X		X	
Patient 30	X		X	
Patient 31	X		X	
Patient 32	X			X
Patient 33	X		X	
Patient 34	X		X	
Patient 35			X	
Patient 36	X			X
Patient 37	X			

Table S4. Primers and probes used for droplet digital PCR.

Primers and Probes	Company	Assay ID	Custom-designed
TERT (c.-146C>T)	Thermo Fisher Scientific	Hs000000093_rm	
TP53 (c.853G>A)	Thermo Fisher Scientific	C_153028087_10	
TERT (c.-124C>T)	IDT		Fd primer seq: CTCCTCCGCGCGGCACCCG<br>Rv primer seq: AAACTCGCGCCGCGAGGA<br>Mutant probe: CAGCCCCTTCCGGGCCCT (HEX)<br>Wt probe: CGACCCCCTCCGGGCCCT (FAM)
ATM (c.1236-2A>T)	IDT		Fd primer seq: TCCTTTTAGTTTGTTAATGTGATGGA<br>Rv primer seq: TCATCAGTAATGGAGACAGCTCA<br>Mutant probe: TGGCTACAGATTGCAACCCA (HEX)

			Wt probe:<br><br>AGGCTACAGATTGCAACCCA (FAM)

SUPPLEMENTARY FIGURES

A)

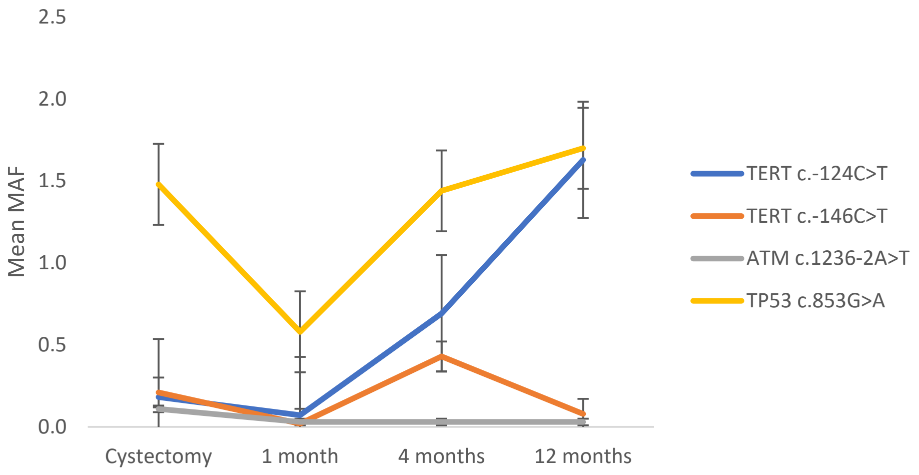

B)

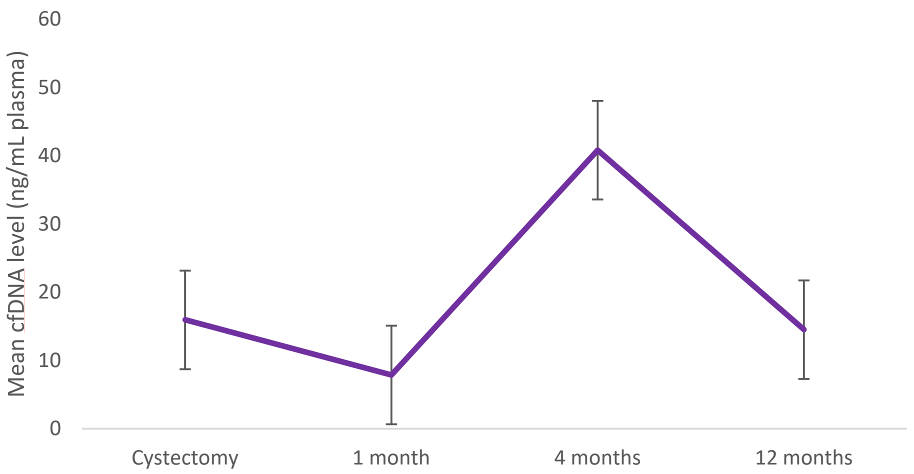

C)

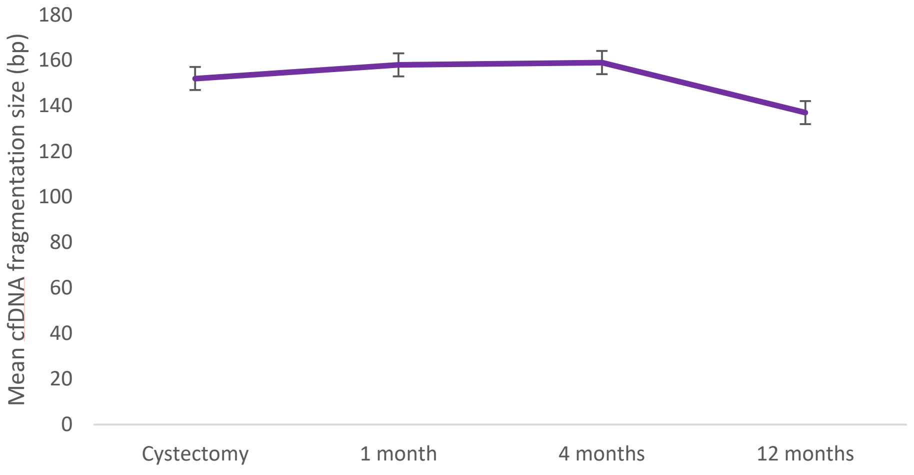

Figure S1. Monitoring of (A) ctDNA status, (B) cfDNA level and (C) cfDNA fragmentation patterns in MIBC patients.

A)

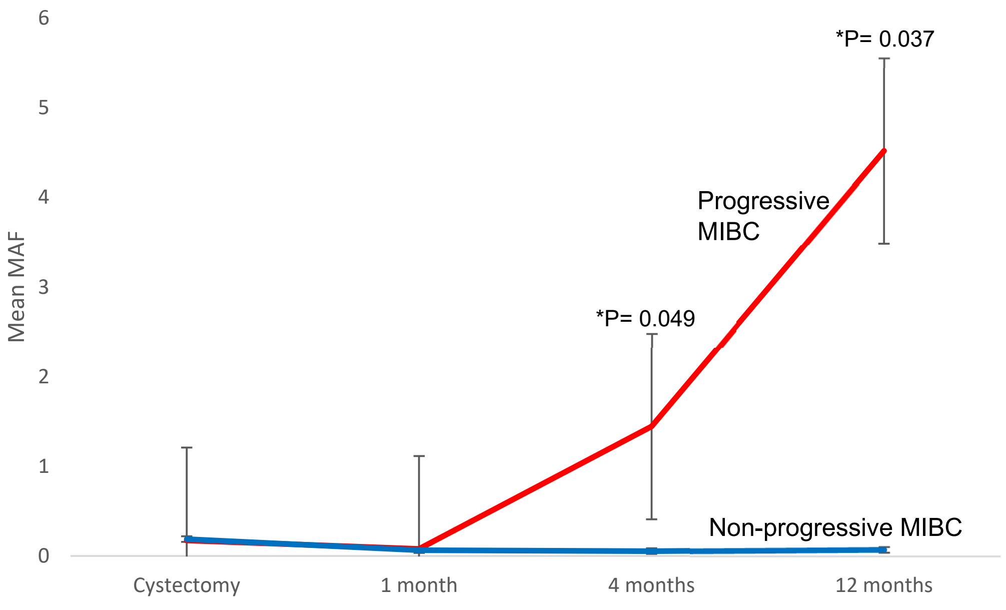

B)

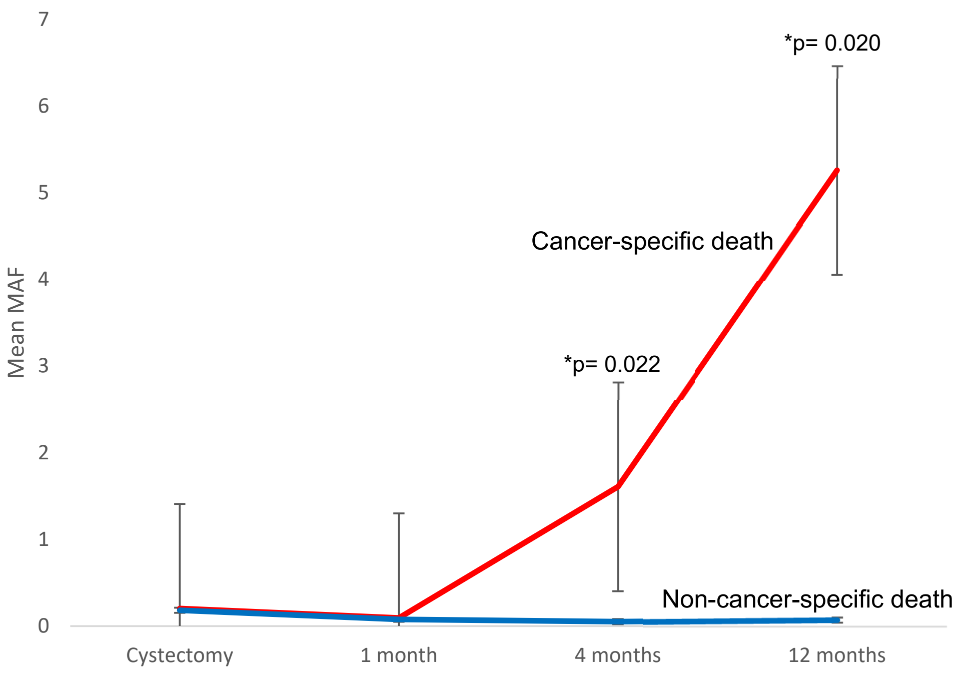

Figure S2. Mutation analysis of *TERT* c.-124C>T comparing MAF in MIBC patients according to (A) tumor progression and (B) death.

A)

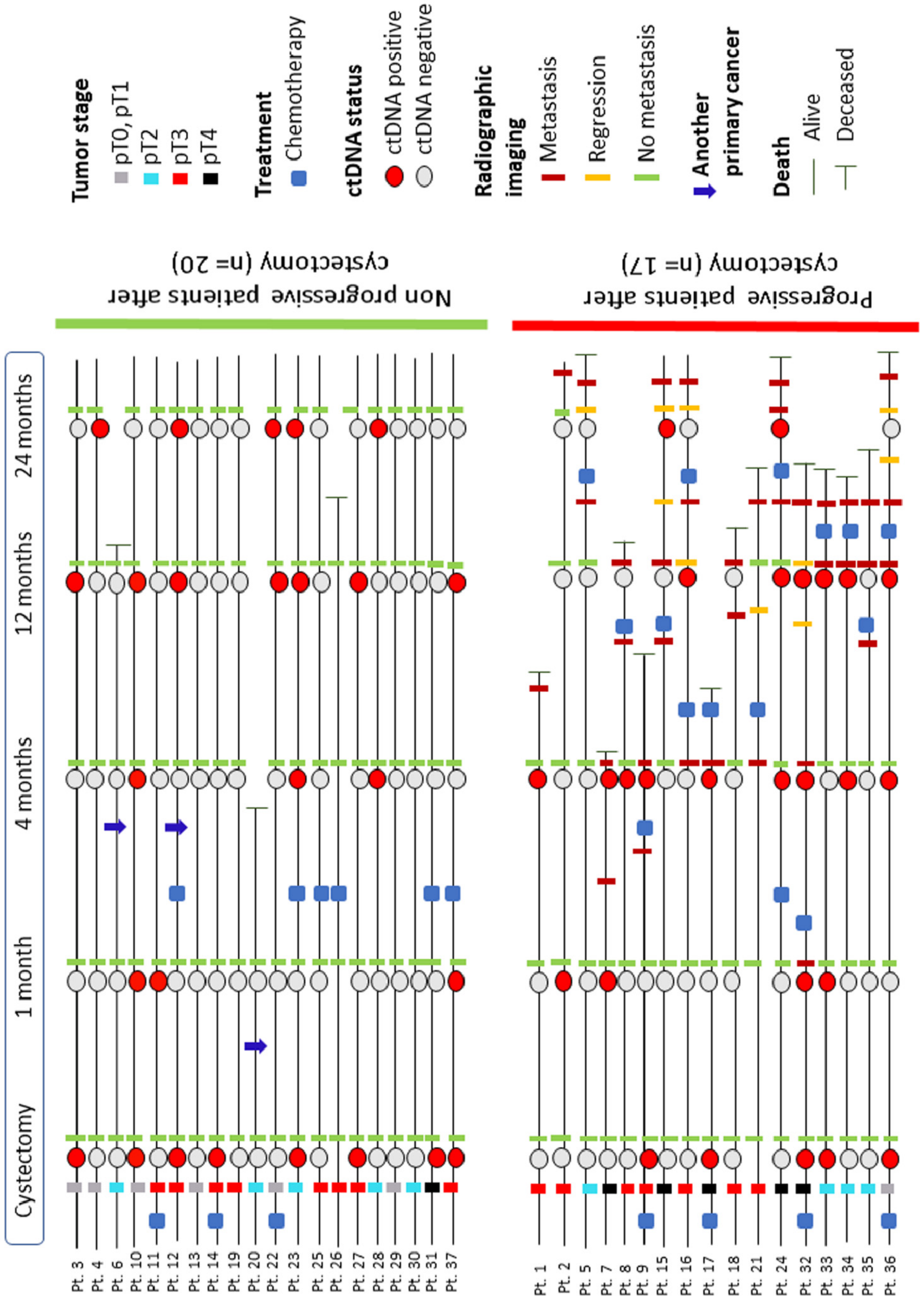

Tumor stage
pT0, pT1
pT2
pT3
pT4
Treatment
Chemotherapy
ctDNA status
ctDNA positive
ctDNA negative
Radiographic imaging
Metastasis
Regression
No metastasis
Another primary cancer
Death
Alive
Deceased
Non progressive patients after cystectomy (n= 20)
Progressive patients after cystectomy (n= 17)
Cystectomy
1 month
4 months
12 months
24 months
Pt. 3
Pt. 4
Pt. 6
Pt. 10
Pt. 11
Pt. 12
Pt. 13
Pt. 14
Pt. 19
Pt. 20
Pt. 22
Pt. 23
Pt. 25
Pt. 26
Pt. 27
Pt. 28
Pt. 29
Pt. 30
Pt. 31
Pt. 37
Pt. 1
Pt. 2
Pt. 5
Pt. 7
Pt. 8
Pt. 9
Pt. 15
Pt. 16
Pt. 17
Pt. 18
Pt. 21
Pt. 24
Pt. 32
Pt. 33
Pt. 34
Pt. 35
Pt. 36

B)

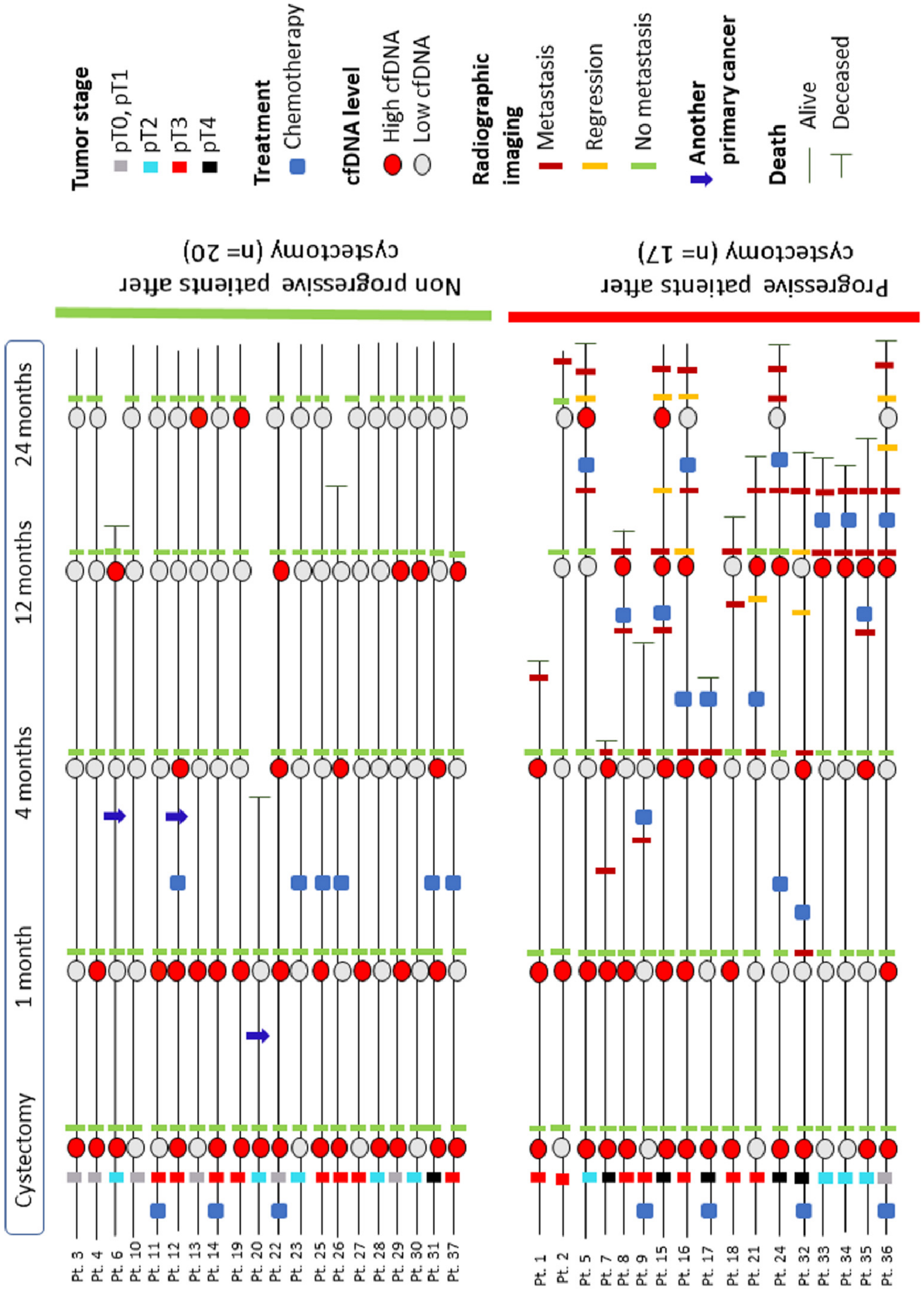

Figure S3. Longitudinal representation of (A) ctDNA status and (B) cfDNA level results for all samples analyzed at different follow-up time points from 37 MIBC patients.

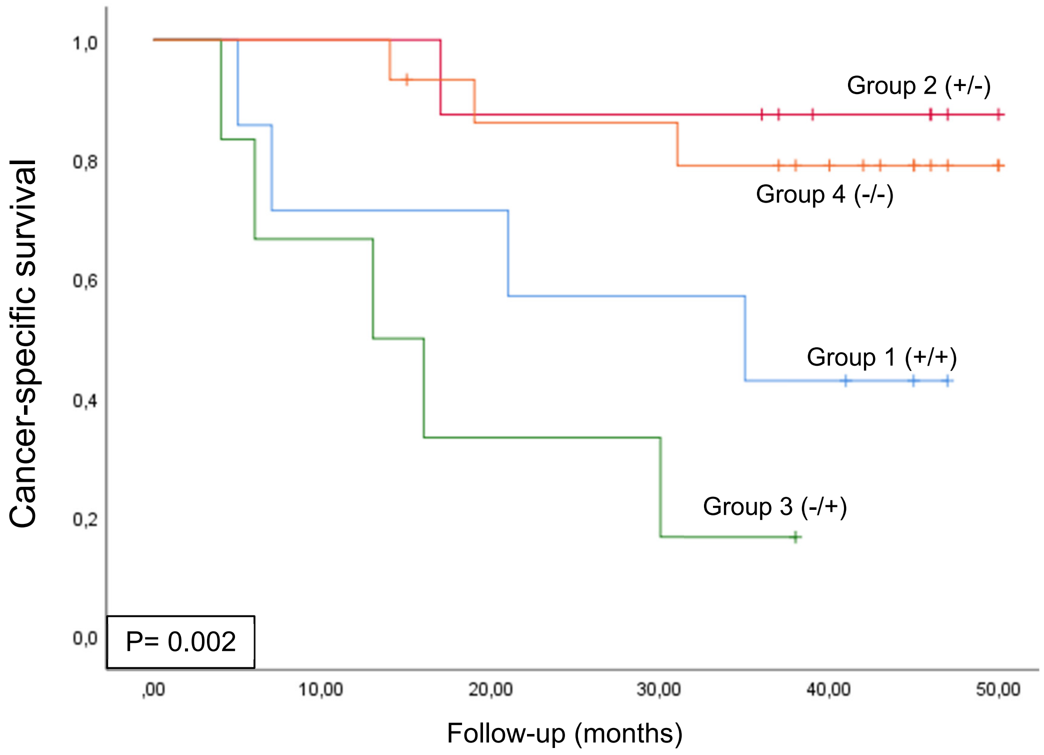

Figure S4. Kaplan-Meier curve for cancer-specific survival in four different MIBC groups stratified according to changes in ctDNA between baseline and four months after RC.

....
